# STUDY OF SECONDARY OSSIFICATION CENTERS OF THE ELBOW IN THE BRAZILIAN POPULATION

**DOI:** 10.1590/1413-785220172506170954

**Published:** 2017

**Authors:** CESAR SATOSHI MIYAZAKI, DANIEL AUGUSTO MARANHO, PAULO MORAES AGNOLLITTO, MARCELLO HENRIQUE NOGUEIRA-BARBOSA

**Affiliations:** 1. Division of Radiology, Ribeirão Preto Medical School, University of São Paulo, Ribeirão Preto, SP, Brazil.; 2. Department of Biomechanics, Medicine, and Rehabilitation of the Locomotor System, Ribeirão Preto Medical School, University of São Paulo, Ribeirão Preto, SP, Brazil.

**Keywords:** Child, Elbow, Radiography, Epiphyses, Growth plate, Growth and development., Criança, Cotovelo, Radiografia, Epífises, Lâmina de crescimento, Crescimento e desenvolvimento.

## Abstract

**Objective:**

To evaluate the age in which the secondary ossification centers of the elbow appear and fuse in the Brazilian population.

**Methods:**

Nearly thirty radiographs were randomly selected for each age group from 0 to 18 years, with a total of 544 radiographs from 439 patients, between 2010 and 2015, without abnormalities secondary to trauma, metabolic or bone tumor diseases. Radiographs were retrospectively evaluated by two blind and independent observers, according to the presence or not of the ossification centers, and the fusion between them.

**Results:**

The age interval of appearance and fusion were, respectively: capitulum (0 to 1 year; 10 to 15 years), radius head (2 to 6 year; 12 to 16 years), medial epicondyle (2 to 8 years; 13 to 17 years), trochlea (5 to 11 years; 10 to 18 years), olecranon (6 to 11 years; 13 to 16 years), e lateral epicondyle (8 to 13 years; 12 to 16 years). Appearance and fusion were earlier in girls compared to boys (exception to capitulum and radius head).

**Conclusion:**

The chronological order was similar to the literature. For girls, the radius head and medial epicondyle appeared simultaneously. There was a tendency of the olecranon center to appear before the trochlea for both sexes. **Level of Evidence III, Diagnostic Study.**

## RESUMO

**Objetivo:**

Avaliar a idade de surgimento e a união dos centros secundários de ossificação do cotovelo na população brasileira.

**Métodos:**

Foram selecionadas aleatoriamente aproximadamente 30 radiografias simples do cotovelo na faixa etária de 0 a 18 anos, no total de 544 radiografias de 439 pacientes, entre 2010 e 2015, sem alterações secundárias a trauma, doença osteometabólica ou tumor. Foram avaliadas retrospectivamente de forma cega e independente por dois observadores, quanto à presença dos centros de ossificação secundária e a união entre eles.

**Resultados:**

O intervalo de idade de aparecimento e de união dos centros foram, respectivamente: capítulo do úmero (0 a 1 ano; 10 a 15 anos), cabeça do rádio (2 a 6 anos; 12 a 16 anos), epicôndilo medial (2 a 8 anos; 13 a 17 anos), tróclea (5 a 11 anos; 10 a 18 anos), olécrano (6 a 11 anos; 13 a 16 anos), e epicôndilo lateral (8 a 13 anos; 12 a 16 anos). No sexo feminino, o aparecimento e união são mais precoces do que no masculino (exceto capítulo do úmero e cabeça do rádio).

**Conclusão:**

A ordem cronológica foi semelhante à da literatura. No sexo feminino, o centro da cabeça do rádio e do epicôndilo medial surgiram simultaneamente. Houve tendência não significativa de o olécrano surgir antes da tróclea em ambos os sexos. **Nível de Evidência III, Estudo Diagnóstico.**

## INTRODUCTION

The bone age evaluation in the skeletally immature patient is important for therapeutic decision-making, and the knowledge about the skeletal development is essential for the results interpretation. The ossification pattern of the secondary centers of the elbow was described in literature,^1^ ^,^ ^2^ and these studies have clinical significance because of the complex radiographic anatomy and associated challenging interpretation for the frequent pediatric cases of trauma.^3^

**Figure 1:**
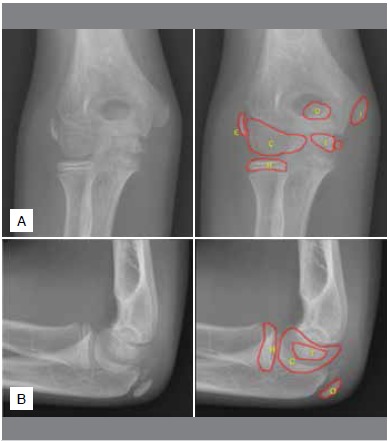
Anterior-posterior (A) and lateral (B) elbow radiographs of a boy with 11 years and six months old. Elbow ossification centers were identified by: C - Capitulum; R - Radius head; I - Medial epicondyle; T - Trochlea; O - Olecranon; E - Lateral epicondyle.

The conventional radiography of the elbow has an intrinsic limitation for evaluating the bone anatomy, considering that the ossification pattern of the cartilaginous component is gradual, fragmented and with contour irregularities. (Figure 1) Some skeletal injuries may not be easily identified in the elbow radiographs. Furthermore, normal radiographic patterns may be misinterpreted as fractures, dislocations, or other abnormalities.^4^ Evaluating the presence or absence of the ossification centers, according to their location and patient’s age, is essential for the diagnosis of traumatic injuries.

The age of appearance of the ossification centers of the pediatric elbow has a relatively well-established chronological sequence in literature: humerus capitulum, radius head, medial or internal epicondyle, humerus trochlea, olecranon, and lateral or external epicondyle. ^4-6^ The mnemonic CRITOE or CRITOL may be applied. The age range for the radiographic appearance of the ossification centers was previously described, however there are some variations that can be associated with differences in ethnic patterns or study methodology.^4^ ^-^ ^6^ Potentially, distinct characteristics in elbow ossification may exist in the Brazilian population, and this information is lacking in the literature. Here, we aimed to evaluate the sequence of appearance and fusion of the ossification centers in radiographs of the pediatric elbow, and correlate with age and sex.

## PATIENTS AND METHODS

This is a retrospective study approved by the Institutional Review Board (11611/2011), with waive of the informed consent. The inclusion criterion was boys and girls with age between zero and eighteen years, who underwent anterior-posterior and lateral elbow radiograph. The exclusion criteria were (1) previous or current elbow fracture; (2) previous surgery, presence of intraosseous orthopaedic implants, or casting apparatus that could compromise the visualization of the ossification centers; (3) suspected or confirmed diagnoses of osteometabolic (e.g. osteogenesis imperfecta), inflammatory (e.g. idiopathic juvenile or piogenic arthritis), bone or soft tissue tumor or any other disorder that could modify the ossification center characteristics, and (4) bad quality radiograph technique (e.g. movement artifacts, inadequate acquisition) or availability of only one incidence.

The patients were allocated in groups according to the age range. Group 0 included new-borns and children aged up to one year; group 1 included patients aged from one to two years, and the same criterion was applied up to 18 years old. Each individual was included in one group only, and for those who were radiographically evaluated more than one time, only the initial exam was considered. We included young adults (18 years) to allow for the inclusion of patients who achieved the skeletal maturity and complete ossification and fusion of the elbow ossification centers.

Initially, we included 926 patients who underwent elbow radiographs between 2010 and 2015. For each age group, we selected approximately 30 patients, using a chronological sequence from the most recent to the oldest exams. The final sample included 544 radiographs from 439 patients (312 boys, 127 girls), with age between 22 days and 18 years. One hundred and five patients were bilaterally evaluated.

The presence or absence of each secondary ossification center (Figure 1) was evaluated following the classification (1) absent; (2) present with no fusion, partial or incomplete fusion; or (3) present with complete fusion. We considered a complete fusion when the growth plate was totally obliterated and ossified.

The imaging evaluation was performed by two radiologists, using a a blind and independent approach without information about age or sex. A second reading was performed following a two-month interval by both observers.

### Statistical analysis

We assessed the inter- and intraobserver agreement using the Kappa coefficient.^7^ Poor reliability is suggested for values between 0 and 0.20; fair reliability from 0.21 to 0.40; moderate reliability from 0.41 to 0.60; substantial or good reliability from 0.61 to 0.80, and almost perfect or very good reliability from 0.81 to 1.0.^8^

A linear regression model with mixed effects (random and fixed effects) was applied to analyze the presence or absence of the ossification centers, and their fusion status, according to patient’s age and sex. The orthogonal contrast test was applied for pos-test estimation. Comparisons among sexes were performed using the Mann-Whitney test. This approach allowed for the estimation of the age of appearance and fusion for boys and girls. The level of significance was set at 5%.

## RESULTS

The intra and interobserver agreement was considered almost perfect for the presence and fusion of all ossification centers. The Kappa coefficient varied between 0.89 e 0.98 for all analysis.

**Figure 2:**
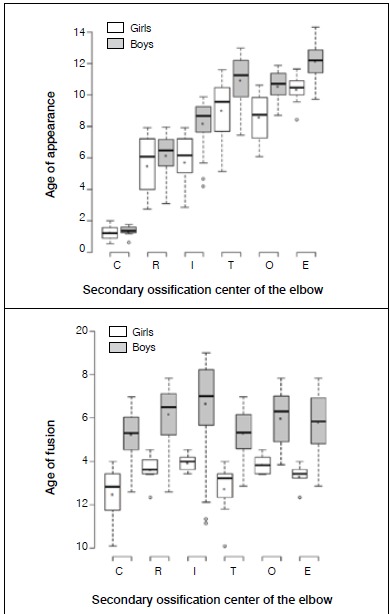
Box plot of age (years) of appearance and fusion of the ossification centers of the elbow. C - Capitulum; R - Radius head; I - Medial epicondyle; T - Trochlea; O - Olecranon; E - Lateral epicondyle.

**Figure 3:**
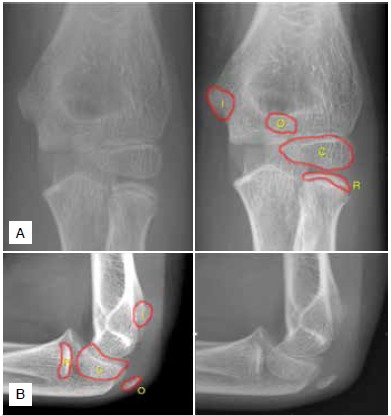
Anterior-posterior (A) and lateral (B) elbow radiographs of a boy with ten years old. It is possible to observe the ossification center of the olecranon without observing the trochlea. C - Capitulum; R - Radius head; I - Medial epicondyle; O - Olecranon.

The first ossification center to appear was the capitulum, around the age one year in both sexes. (Table 1, Figure 2) In girls, the ossification center of the radius head and the medial epicondyle appeared at the same age (median, 6.1 years). In contrast, we observed that the ossification center of the radius head appeared earlier (median, 6.5 years) than the medial epicondyle (median, 8.7 years) in boys. (Table 1 and Figure 2) Although we did not observe significant difference, there was a tendency for the olecranon to ossify earlier than the trochlea in girls and boys, at a median of 8.7 and 10.7 years (olecranon) versus 9.6 and 11.3 years (trochlea) (Table 2 and Figure 3). The estimated difference was 0.39 years in girls (95% confidence interval [95%IC] -0.31 - 1.09, p=0.27) and 0.23 years in boys (95%IC -0.25-0.71, p=0.34). Table 2 describes the estimated differences for the age of appearance between boys and girls.

**Table 1:** Age (in years) of appearance and fusion of the elbow ossification centers for boys and girls.

		Age of appearance of the ossification centers (mean ± standard deviation; years)					Age of fusion of the ossification centers (mean ± standard deviation; years)			
Center	n	Girls	n	Boys	p	n	Girls	n	Boys	p
C	19	1.26 ± 0.45	9	1.36 ± 0.36	<0.01	11	12.50 ± 1.22	59	15.25 ± 1.05	<0.01
R	30	5.52 ± 1.60	53	6.19 ± 1.27	<0.01	7	13.64 ± 0.71	39	16.19 ± 1.28	<0.01
I	24	5.75 ± 1.60	53	8.21 ± 1.36	<0.01	5	13.95 ± 0.43	64	16.69 ± 1.90	<0.01
T	19	9.06 ± 1.86	56	10.98 ± 1.44	<0.01	9	12.75 ± 1.20	55	15.32 ± 1.01	<0.01
O	23	8.60 ± 1.40	38	10.59 ± 0.87	<0.01	6	13.86 ± 0.45	47	16.01 ± 1.21	<0.01
E	11	10.36 ± 0.89	46	12.18 ± 1.12	<0.01	6	13.33 ± 0.55	57	15.82 ± 1.23	<0.01

C - Capitulum; R - Radius head; I - Medial epicondyle; T - Trochlea; O - Olecranon; E - Lateral epicondyle. *P-value refers to the comparison between boys and girls.

**Table 2:** Estimated difference (years) in the age of appearance of the elbow secondary ossification centers between boys and girls.

Center	Estimated difference between boys and girls	95% confidence interval		p-value*
C	0.10	-0.96	1.17	0.85
R	0.70	0.11	1.29	0.02
I	2.45	1.83	3.08	<0.01
T	2.16	1.48	2.84	<0.01
O	2.32	1.65	2.99	<0.01
E	2.19	1.34	3.04	<0.01

C - Capitulum; R - Radius head; I - Medial epicondyle; T - Trochlea; O - Olecranon; E - Lateral epicondyle. * p-value refers to the comparison between boys and girls.

All the secondary ossification centers of the elbow presented with a tendency to show a complete fusion at earlier ages in girls compared to boys. (Table 1 and Figure 2)

## DISCUSSION

The evaluation of the bone age is an important tool for several therapeutic decision-makings, including orthopaedic conditions such as scoliosis and lower limb asymmetry in skeletal immature patients. In Endocrinology, the bone age is routinely assessed in the suspicion of precocious puberty. The bone age may be estimated using several techniques for different anatomic regions, for example the hand, pelvis, foot, knee and elbow.

A classical example is the Risser classification, which evaluate the potential for growth during the scoliosis treatment planning.^9^ Other clinically relevant method for bone age assessment is the Greulich and Pyle,^10^ using posterior-anterior hand and wrist radiographs.

In 1962, Sauvegrain et al.^11^ evaluated anterior-posterior radiographs of the elbow for the bone age assessment in children and adolescents. They evaluated the lateral epicondyle, trochlea, olecranon and radius head, based on the shape and development of these ossification centers. A grading system was compared to a graph that correlates the estimated bone age with the puberty evaluation and pre-puberal stage after age 10 years.

Evidence has been reported in literature on the age of appearance and fusion of the secondary ossification centers of the elbow ,^1^ ^,^ ^2^ ^,^ ^5^ ^,^ ^12^ ^,^ ^13^ (Table 3) however small population samples and incomplete information regarding methodology may decrease the generalizability.

**Table 3:** Age (years) of appearance of the secondary ossification centers of the elbow in boys and girls, according to different studies in literature.

Center	Girdany and Golden^1^		Garn et al.^2^		Cheng et al.^5^		Patel et al.^12^		Bajaj et al.^13^	
	♀	♂	♀	♂	♀	♂	♀	♂	♀	♂
C	0.3	0.1-0.7	1	0.3		1			0.5	0.5
R	5.2	2.9-5.5	7	3.9	5.9	5	4.2	5.9	3.5	6.2
I	2.3 - 5.1	4.7-5.7	3.4		5	7	4.2	6.8	5	7.4
T		7 - 9	11	6.3	9.7	9	8.4	9.7	7.7	7.9
O	9.7	8 - 11	11	8.0	9.9	9	8.3	9.9	8.6	10.4
E	11.2	11 - 14	12	9.2	11.2	10	9.4	11.2	7.5	10.2

C - Capitulum; R - Radius head; I - Medial epicondyle; T - Trochlea; O - Olecranon; E - Lateral epicondyle.

The methodology used in our study was similar to the study from Cheng et al.,^5^ who evaluated the elbow ossification center in the Chinese population. We added the differences among sexes, similarly to the methodology of Patel et al.,^12^ who evaluated the age of fusion of the ossification centers of the elbow in the Canadian population. 

We identified a mean difference of approximately two years in the age of appearance of the ossification centers between girls and boys, and this difference is in line with the studies from Cheng et al.^5^ e Patel et al.^12^ However, the difference was smaller for capitulum and the radius head. For the age of fusion of the ossification centers, we did not observe a clear sequence compared to the age of appearance. Nevertheless, girls had an age of fusion significantly smaller than boys, for all ossification centers.

Ossification patterns may be influenced by genetic and environmental factors, as well as other conditions that can affect the skeletal growth and maturity. The comparison with the population from India,^13^ China^5^ and Canada^12^ confirmed probable regional differences, which may explain some variations in these studies.

The secondary ossification centers of the elbow may present physiological multicentric and fragmentation aspect. Determining the chronological sequence of appearance and their physiological characteristics plays an important role in the pediatric trauma evaluation. The differential diagnosis between fractures, growth plate injuries and normal radiographic variations is challenging.

Some study limitations must be cited. During the patient allocation, we could not match patients by sex, because trauma was much more common in boys than in girls. As consequence, our sample had a greater number of boys. There was no longitudinal and controlled radiographic evaluation of our patients, therefore we could not evaluate the sequence of appearance of the ossification centers using a longitudinal methodology. However, we estimated the chronological sequence using the prevalence by age group. We observed some discrepancies because of a low number of girls in the groups five years (low prevalence of the capitulum presence) and nine years (low prevalence of the trochlea presence). Nevertheless, high reliability was observed by means of Kappa coefficient between observers.

## CONCLUSION

The olecranon center showed a tendency to ossify earlier than the trochlea center in girls and boys, although we did not find significant difference with our sample size. The radius head and medial epicondyle centers appeared simultaneously in girls. In general, the ossification centers appear two years earlier in girls compared to boys, except for the capitulum and radius head. Girls were younger when the ossification center showed complete fusion, however we could not observe a clear chronologic sequence of fusion. Our results showed that the secondary ossification centers of the elbow appear sequentially with a chronologic order in the Brazilian population, that is similar to the orders previously described.
